# Comparative study of esophagectomy, endoscopic therapy, and radiotherapy for cT1N0M0 esophageal cancer in elderly patients: A SEER database analysis

**DOI:** 10.1111/1759-7714.13080

**Published:** 2019-06-14

**Authors:** Jianjun Qin, Yinjie Peng, Weipeng Chen, Haibo Ma, Yan Zheng, Yin Li, Jun Wang

**Affiliations:** ^1^ Department of Thoracic Surgery, National Cancer Center/National Clinical Research Center for Cancer/Cancer Hospital Chinese Academy of Medical Sciences & Peking Union Medical College Beijing China; ^2^ Department of Thoracic Surgery The Affiliated Cancer Hospital of Zhengzhou University Zhengzhou China; ^3^ Department of Radiation Oncology Fourth Hospital of Hebei Medical University Shijiazhuang China

**Keywords:** endoscopic therapy, esophageal cancer, esophagectomy, radiotherapy

## Abstract

**Background:**

The number of patients diagnosed with early stage disease (T1a or T1b) has been increasing. This study was conducted to investigate the effect of esophagectomy (ES), endoscopic therapy (ET), and radiotherapy (RT) on long‐term survival in elderly patients with cT1N0M0 esophageal cancer.

**Methods:**

We searched the Surveillance, Epidemiology, and End Results (SEER) database to identify the records of elderly patients (≥ 75 years) with cT1N0M0 esophageal cancer between 2004 and 2014. Patient demographics and esophageal cancer parameters were compared among ES, ET, and RT groups. The Kaplan–Meier method and Cox proportional hazard modeling were used to compare long‐term survival.

**Results:**

Data from 954 esophageal cancer patients (ES: *n* = 196; ET: *n* = 224; RT: *n* = 534) were identified. Multivariate Cox regression analysis showed that five‐year survival in the ET and ES groups was significantly higher than in the RT group. After propensity score matching, we found no difference in five‐year survival between ES and ET.

**Conclusion:**

Using SEER data, we identified a significant survival advantage with the use of ES and ET compared to RT in patients with cT1N0M0 esophageal cancer aged > 75 years, while the long‐term survival of patients after ET and ES was not significantly different.

## Introduction

Esophageal cancer is a common digestive cancer and the sixth leading cause of cancer death worldwide, with more than 450 000 diagnoses and 400 000 deaths in 2012.^1^, ^2^ Although diagnostic techniques and treatment of esophageal cancer has improved over the last few decades, the overall five‐year survival rate is still only 15–25%.^3^, ^4^ Because of an awareness of early lesions by endoscopists, early stage cancer patients are now seen more frequently. Overall, approximately 20% of all resected esophageal cancers are early lesions limited to the mucosa and submucosa.^2^

The National Comprehensive Cancer Network (NCCN) guidelines recommend the following treatment for early esophageal cancer: in patients with Tis and T1a esophageal cancer, endoscopic therapy (ET) is the preferred therapeutic approach; in patients with T1b esophageal cancer, esophagectomy (ES) is currently the preferred therapy or ET may be an alternative strategy to ES, especially in patients who are poor surgical candidates.

Esophagectomy remains the cornerstone of treatment for resectable esophageal carcinoma.^5^, ^6^ However, surgery is a highly invasive procedure that can lead to recurrence and metastasis in 60% of cases,^1^, ^7^ over 5% postoperative mortality,^2^ and more complications than ET.^8^, ^9^ Recent advances in radiotherapy (RT) have shown promise for improving outcomes and survival, and decreasing recurrence and metastasis^4^, ^10^ however, the outcome of RT remains unsatisfactory in terms of lung and heart toxicity, and survival in elderly cancer patients is impaired by comorbidities and reduced performance status^11^ Recently, ET (endoscopic polypectomy or mucosal resection, photodynamic therapy, radiofrequency ablation, and freezing treatment) has become widely accepted for the treatment of early esophageal cancer^12^, ^13^ However, ET might result in inadequate resection or staging because of the absence of lymphadenectomy. Therefore, guidelines for the management of early esophageal cancer are essential. Most of the published studies do not compare overall survival (OS) or cancer‐specific survival (CSS) among ES, ET, and RT, partially because the heterogeneity of cT1‐2N0M0 esophageal cancer patients excluded them from many phase III trials of trimodality therapy.^14^

The American Geriatrics Society defines elderly as an age of ≥ 75 years.^15^, ^16^ The median age of esophageal cancer is 68 years, with more than 30% of patients aged > 75. There has been controversy over the best treatment for early esophageal cancer in the elderly. Treatment efficacy and tolerance in elderly patients with early esophageal cancer (cT1N0M0) is potentially impacted by a variety of conditions, such as life expectancy, quality of life, peri‐operative complications, and willingness to undergo treatment. In some cases, chronological age does not accurately predict tolerance to chemotherapy or RT.^4^ Because of the lack of clinical trials, we are currently limited to the results of small retrospective and single center studies.^9^ Therefore, we used the Surveillance, Epidemiology, and End Results (SEER) database to conduct a national descriptive epidemiological study to compare the effect of ES, ET, and RT on long‐term survival in elderly patients with cT1N0M0 esophageal cancer.

## Methods

### Data source

A retrospective study was performed using data from the SEER database for the years 2004–2014. The SEER database is derived from 18 cancer registries representing approximately 28% of the United States.^17^, ^18^ Patients aged ≥ 75 years with cT1N0M0 esophageal cancer were included. Tumor node metastasis (TNM) staging was identified according to the 6th edition American Joint Committee on Cancer (AJCC) TNM system. Patients with missing treatment data or unknown survival status were excluded. Patient demographics and cancer characteristics including age; gender; race (white, black, other/unknown); marital status (married, other/unknown); cause of death (alive, esophageal cancer, other); tumor differentiation (high/moderate grade, poorly/undifferentiated, unknown); and histological type (adenocarcinoma, squamous cell carcinoma, other) were collected.

### Study population

Patients were divided into three groups according to the treatment modality: ET, ES and RT. ET consisted of local tumor destruction (photodynamic therapy, cryotherapy, laser ablation) and/or excision (polypectomy or mucosal resection, excision biopsy, laser resection) via an endoscopic approach, which was not differentiated in the database. ES was defined as any form of esophageal resection including partial or total removal of the esophagus, partial or total removal combined with partial or total removal gastrectomy, or partial or total esophagectomy combined with laryngectomy. RT was therapy using ionizing radiation. Patients were divided into younger (75–79 years) and older (≥ 80 years) groups, and stratified by year of diagnosis as early (2004–2009) or late (year of diagnosis 2010–2014).

### Statistical analysis

All patients in the unmatched dataset met the inclusion criteria. Propensity score matching (PSM) can help to achieve balanced covariates across treatment groups. Patients in the two groups were matched 1:1 using the nearest propensity score (PS) on the logit scale. A matched dataset was created using PS of age, gender, race, tumor differentiation, histological type, and year of diagnosis. After PSM, differences in categorical clinical characteristics were tested for significance by chi‐square tests.

Five‐year OS and CSS were calculated and expressed as months. The OS was right censored if the patient was alive at study termination or was lost to follow‐up, and patient death was considered an event. In CSS analysis, surviving patients or those that died from other reasons were censored, while death from esophageal cancer was considered an event. The Kaplan–Meier method was used to generate the survival curve. A log‐rank test was performed to compare OS and CSS among ES, ET, and RT groups. A multivariate Cox proportional hazards model was constructed to assess the hazard ratios (HRs) and 95% confidence intervals (CI) of eight covariates: age, gender, race, marital status, T staging, tumor differentiation, histological type, and year of diagnosis. SPSS version 22.0 was used for statistical analyses. All tests were two‐sided with a significance level of *P* < 0.05.

## Results

### Demographics and trend

The SEER database included data of 954 patients (ES: *n* = 196, 20.5%; ET: *n* = 224, 23.5%; RT: *n* = 534, 56%) that met our study criteria. The patient demographics and tumor parameters are listed in Table 1. Endoscopic local tumor resection was the most common type of ET performed (Table 2). The median ages of the ES, ET, and RT groups were 77, 80, and 82 years, respectively (*P* < 0.001). Patients in the ES group were younger than those in the ET and RT groups (Table 3). Trends in the use of ES, ET, and RT over time are shown in Figure 2. There was an increase in the proportion of patients who underwent ET from the year of diagnosis in 2004–2009 (19.1%) to 2010–2014 (30.1%). The number of cases treated via ES remained relatively stable (20.5% vs. 20.7%) over the two time periods, while the number of cases treated via RT decreased from 60.5% to 49.2% (*P* < 0.001).

**Table 1:** Baseline and tumor characteristics

Characteristic	Esophagectomy (*n* = 196)	Endoscopic therapy (*n* = 224)	Radiotherapy (*n* = 534)	*P*
Age (years)				
Early (75–79)	140 (71.4%)	104 (46.4%)	199 (37.3%)	< 0.001
Elderly (> 79)	56 (28.6%)	120 (53.6%)	335 (62.7%)	
Gender				
Male	156 (79.6%)	174 (77.7%)	348 (65.2%)	< 0.001
Female	40 (20.4%)	50 (22.3%)	186 (34.8%)	
Race				
White	178 (90.8%)	212 (94.6%)	465 (87.1%)	
Black	3 (1.5%)	6 (2.7%)	38 (7.1%)	0.01
Other/unknown	15 (7.7%)	6(2.7%)	31 (5.8%)	
Marital status				
Married	127 (64.8%)	140 (62.5%)	272 (50.9%)	< 0.001
Other/unknown	69 (35.2%)	84 (37.5%)	262 (49.1)	
Tumor grade				
G1/2 (well/moderate)	108 (55.1%)	101 (45.1%)	240 (44.9%)	< 0.001
G3/4 (poor/undifferentiated)	56 (28.6%)	36 (16.1%)	190 (35.6%)	
Unknown	32 (16.3%)	87 (38.8%)	104 (19.5%)	
Histology				
Squamous cell carcinoma	38 (19.4%)	24 (10.7%)	223 (41.8%)	< 0.001
Adenocarcinoma	156 (79.6%)	184 (82.1%)	283 (53.0%)	
Unknown	2 (1%)	16 (7.1%)	28 (5.2%)	
T stage				
T1a	47 (24.0%)	52 (23.2%)	33 (6.2%)	< 0.001
T1b	94 (48.0%)	43 (19.2)	24 (4.5%)	
T1x	55 (28.1%)	129 (57.6%)	477 (89.3%)	
Cause of death				
Alive	116 (59.2%)	127 (56.7%)	113 (21.2%)	< 0.001
Esophagus	42 (21.4%)	36 (16.1%)	309 (57.9%)	
Other cause of death	38 (19.4%)	61 (27.2)	112 (21.0%)	
Time period				
Early (2004–2009)	117 (59.7%)	109 (48.7%)	346 (64.8%)	< 0.001
Late (2010–2014)	79 (40.3%)	115 (51.3%)	188 (35.2%)	

**Table 2:** Distribution of endoscopic therapy

Procedure	Overall N (%)	Early (2004–2009) N (%)	Late (2010–2014) N (%)
Local tumor destruction			
Photodynamic therapy	9 (4.0)	9 (8.3)	0
Cryosurgery	6 (2.7)	4 (3.7)	2 (1.7)
Laser	5 (2.2)	3 (2.8)	2 (1.7)
NOS	6 (2.7)	2 (1.8)	4 (3.5)
Local tumor excision			
Polypectomy	16 (7.1)	7 (6.4)	9 (7.8)
Excisional biopsy	96 (42.9)	42 (38.5)	54 (47.0)
Laser excision	2 (0.9)	1 (0.9)	1 (0.9)
NOS	46 (20.5)	23 (21.1)	23 (20.0)
Combined local tumor destruction and excision	38 (17.0)	18 (16.5)	20 (17.4)

NOS, not otherwise specified.

**Table 3:** Characteristics of patients treated with ET and ES for early‐stage esophageal cancer

	Before matching			After matching		
	ES	ET		ES	ET	
Characteristic	(*n* = 196)	(*n* = 224)	*P*	(*n* = 70)	(*n* = 70)	*P*
Age (years)						
Elderly (75–79)	140 (71.4%)	104 (46.4%)	< 0.001	49 (70%)	45 (64.3%)	0.472
Early (> 79)	56 (28.6%)	120 (53.6%)		21 (30%)	25 (35.7%)	
Gender						
Male	156 (79.6%)	174 (77.7%)	0.72	61 (87.1%)	60 (85.7%)	0.805
Female	40 (20.4%)	50 (22.3%)		9 (12.9%)	10 (14.3%)	
Race						
White	178 (90.8%)	212 (94.6%)		64 (91.4%)	65 (92.9%)	0.927
Black	3 (1.5%)	6 (2.7%)	0.043	2 (2.9%)	2 (2.9%)	
Other/unknown	15 (7.7%)	6 (2.7%)		4 (5.7%)	3 (4.3%)	
Marital status						
Married	127 (64.8%)	140 (62.5%)	0.69	48 (68.6%)	44 (62.9%)	0.476
Other/unknown	69 (35.2%)	84 (37.5%)		22 (31.4%)	26 (37.1%)	
Tumor grade						
G1/2 (well/moderate)	108 (55.1%)	101 (45.1%)	< 0.001	41 (58.6%)	41 (58.6%)	0.557
G3/4 (poor/undifferentiated)	56 (28.6%)	36(16.1%)		20 (28.6%)	16 (22.9%)	
Unknown	32 (16.3%)	87 (38.8%)		9 (12.9%)	13 (18.6%)	
Histology						
Squamous cell carcinoma	38 (19.4%)	24 (10.7%)	0.001	8 (11.4%)	11 (15.7%)	0.448
Adenocarcinoma	156 (79.6%)	184 (82.1%)		62 (88.6%)	58 (82.9%)	
Unknown	2 (1%)	16 (7.1%)		0 (0.0%)	1 (1.4%)	
T stage						
T1a	47 (24.0%)	52 (23.2%)	< 0.001	17 (24.3%)	17 (24.3%)	< 0.001
T1b	94 (48.0%)	43 (19.2)		36 (51.4%)	15 (21.4%)	
T1x	55 (28.1%)	129 (57.6%)		17 (24.3%)	38 (54.3%)	
Cause of death						
Alive	154 (78.6%)	188 (83.9%)	0.17	55 (78.6%)	55 (78.6%)	1
Esophagus/other cause of death	42 (21.4%)	36 (16.1%)		15 (21.4%)	15 (21.4%)	
Time period						
Early (2004–2009)	117 (51.8%)	109 (48.2%)	0.025	37 (52.9%)	40 (57.1%)	0.61
Late (2010–2014)	79(40.7%)	115(59.3%)		33(47.1%)	30(42.9%)	

ES, esophagectomy; ET, endoscopic therapy.

### Survival analysis

**Figure 1:**
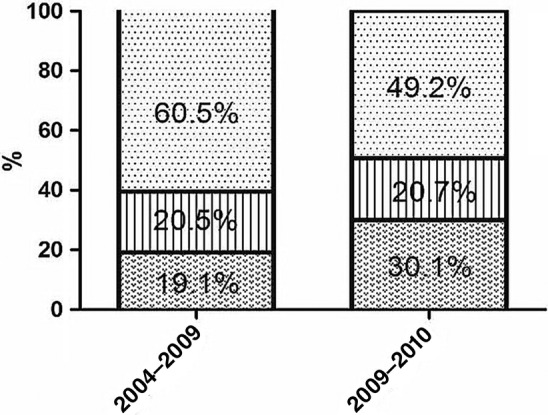
Changes in use of esophagectomy (ES), endoscopic therapy (ET), and radiotherapy (RT) from 2004–2009 to 2010–2014 (*P* < 0.001). (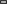) ET, (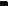) ES and (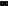) RT.

OS between the ET and RT groups differed significantly, with a median survival time of 50 months (95% CI 41–59) in the ET group versus 15 months (95% CI 13.4–16.6) in the RT group (*P* < 0.001). The survival curves for the three treatment groups are shown in Figure 1. The five‐year OS rates in the ES, ET, and RT groups were 50.5%, 39.6%, and 12.1%, respectively (*P* < 0.001), while CSS rates were 70.3%, 72.2%, and 24.7%, respectively (*P* < 0.001). There was also an increase in five‐year OS (24.1 vs. 28%; *P* = 0.005) and CSS (42.9 vs. 53.2%; *P* = 0.02) from 2004–2009 to 2010–2014.

**Figure 2:**
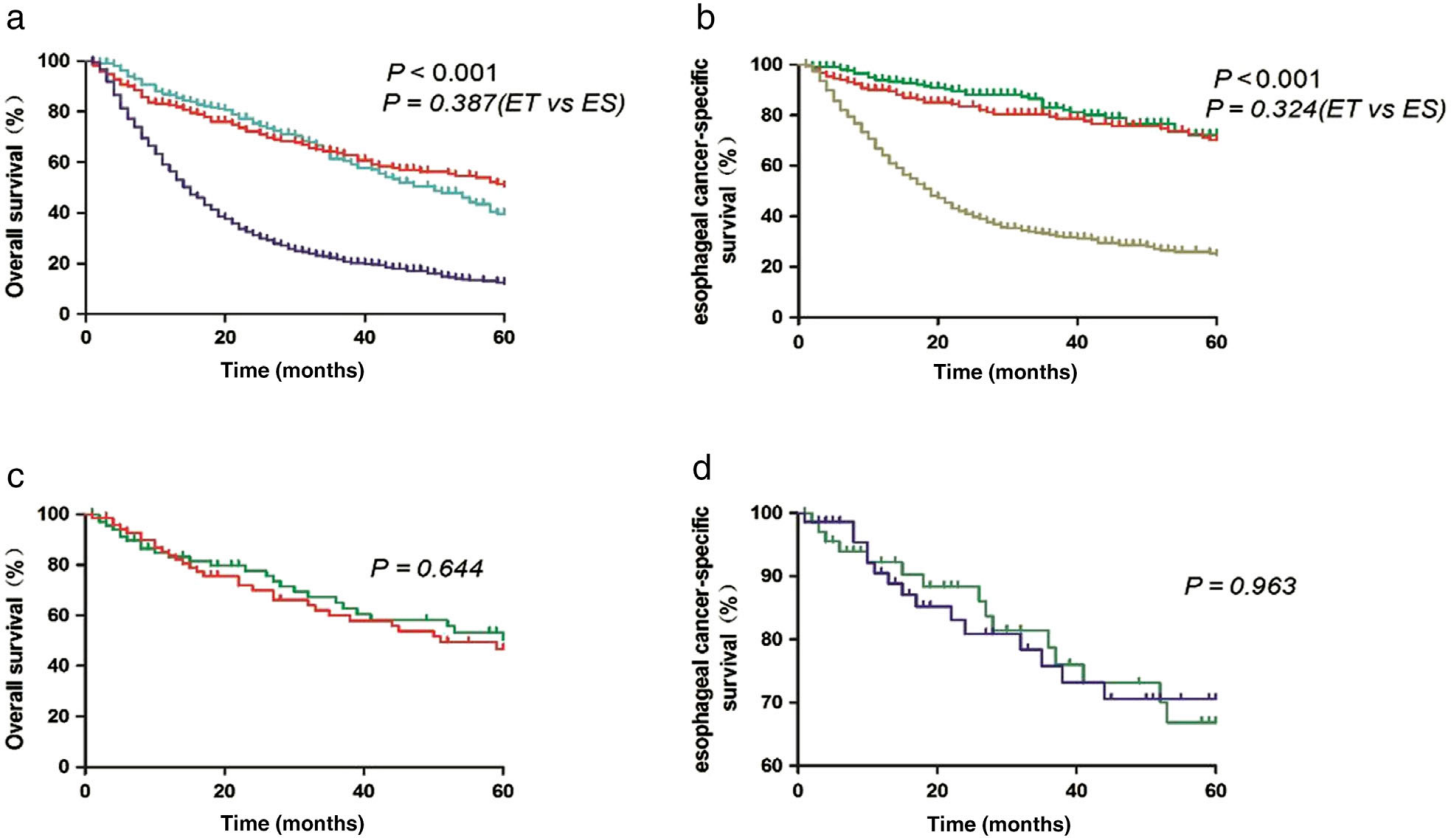
(**a**) Overall survival (OS) and (**b**) esophageal cancer‐specific survival (CSS) rates in patients with early esophageal cancer undergoing esophagectomy (ES), endoscopic treatment (ET), or radiotherapy (RT). (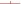) Endoscopic therapy, (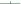) Esophagectomy and (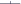) Radiotherapy. (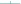) Endoscopic therapy, (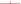) Esophagectomy and (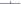) Radiotherapy. (**c**) OS and (**d**) esophageal CSS in patients with early esophageal cancer undergoing ES or ET after propensity score matching. (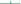) Esophagectomy and (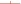) Endoscopic therapy. (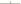) Esophagectomy and (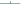) Endoscopic therapy.

The five‐year OS (ET: HR 0.371, 95% CI 0.291–0.473, *P* < 0.001; ES: HR 0.305, 95% CI: 0.227–0.408, *P* < 0.001) and CSS (ET: HR 0.213, 95% CI 0.147–0.309, *P* < 0.001; ES: HR 0.246, 95% CI 0.166–0.364, *P* < 0.001) rates in the ET and ES groups were significantly higher than in the RT group. Compared to ET, patients treated with ES had similar OS (HR 0.822, 95% CI 0.600–1.126; *P* = 0.222) and CSS (HR 1.152, 95% CI 0.722–1.838; *P* = 0.553). The T1a patients in the ES group had higher five‐year OS (65.1% vs. 37.3%; *P* = 0.031) and similar CSS (78.9% vs. 65.3%, *P* = 0.241) compared to patients in the ET group, but the T1b patients in the ET group had higher five‐year CSS (82.3% vs. 53.8%, *P* = 0.049) and similar OS (33.6% vs. 35.2%, *P* = 0.786) compared to those in the ES group.

PSM produced 70 patient pairs and the patient characteristics and cancer‐related variables of both treatment groups after propensity matching are shown in Table 3. Survival analysis and log‐rank testing showed similar OS and CSS rates in the ET and ES groups (Fig 1). Subgroup analyses showed no difference in five‐year OS and CSS between the ES and ET groups with T1a (OS: 59% vs. 28.1%, *P* = 0.084; CSS: 75.5% vs. 68.8%, *P* = 0.558) and T1b (OS: 39.1% vs. 35.9%, *P* = 0.725; CSS: 54.9% vs. 75.4%, *P* = 0.872) staging. In addition, after PSM (Table 4), Cox proportional hazards regression revealed that ES did not improve five‐year OS (HR 0.756, 95% CI 0.419–1.366; *P* = 0.354) or CSS (HR 0.89, 95% CI 0.394–2.013; *P* = 0.78) compared to ET.

**Table 4:** Multivariate analyses of OS and CSS in patients with early esophageal cancer undergoing ES or ET after propensity score matching

	OS		CSS	
Variable	HR (95% CI)	*P*	HR (95% CI)	*P*
Treatment group				
ET	1		1	
ES	0.756 (0.419–1.366)	0.354	0.890 (0.394–2.013)	0.78
Age (years)				
Early (75–79)	1		1	
Elderly (> 79)	1.689 (0.954–2.991)	0.072	2.306 (1.036–5.137)	0.0401
Gender				
Female	1		1	
Male	2.086 (0.767–5.672)	0.15	4.175 (0.932–18.692)	0.062
Race		0.551		0.346
White	1		1	
Black	0.729 (0.098–5.418)	0.757	1.612 (0.203–12.778)	0.651
Other/unknown	0.491 (0.132–1.834)	0.29	0.299 (0.054–1.672)	0.169
Marital status				
Single/other	1		1	
Married	0.728 (0.385–1.377)	0.329	0.637 (0.258–1.571)	0.327
Tumor grade		0.739		0.292
G1/2 (well/moderate)	1		1	
G3/4 (poor/undifferentiated)	1.259 (0.675–2.351)	0.469	1.973 (0.842–4.626)	0.118
Unknown	0.957 (0.425–2.238)	0.953	1.504 (0.485–4.667)	0.48
Histology		0.023		0.011
Squamous cell carcinoma	1		1	
Adenocarcinoma	0.395 (0.161–0.969)	0.043	0.165 (0.051–0.533)	0.003
Unknown	3.589 (0.366–35.222)	0.273	0	0.984
T stage		0.195		0.509
T1a	1		1	
T1b	1.612 (0.780–3.331)	0.197	1.729 (0.587–5.089)	0.32
T1x	0.845 (0.394–1.814)	0.666	1.028 (0.351–3.013)	0.96
Time period				
Early (2004–2009)	1		1	
Late (2010–2014)	0.686 (0.360–1.306)	0.251	0.368 (0.135–1.002)	0.051

CI, confidence interval; CSS, cancer‐specific survival; ES, esophagectomy; ET, endoscopic therapy; HR, hazard ratio; OS, overall survival.

Univariate and multivariate Cox regression analyses were performed for the whole cohort. Univariate analysis revealed that treatment method, age, marital status, T staging, tumor differentiation, histological type, and year of diagnosis were independently associated with five‐year OS, while treatment method, age, gender, marital status, T staging, tumor differentiation, histological type, and year of diagnosis were independently associated with CSS (Table 5). Multivariate Cox regression analysis revealed that radiotherapy, older age at diagnosis, low grade or undifferentiated, T1b staging, unmarried, and early year of diagnosis were independent risk predictors for five‐year OS, while RT, older age at diagnosis, low grade or undifferentiated, and T1b staging were independent risk predictors for CSS (Table 6).

**Table 5:** Univariable analysis of predictors of OS and CSS

	OS		CSS	
Variable	HR (95% CI)	*P*	HR (95% CI)	*P*
Treatment group		< 0.001		< 0.001
RT	1		1	
ET	0.349 (0.279–0.435)	< 0.001	0.184 (0.130–0.260)	< 0.001
ES	0.313 (0.245–0.398)	< 0.001	0.237 (0.171–0.328)	< 0.001
Age (years)				
Early (75–79)	1		1	
Elderly (> 79)	1.552 (1.318–1.827)	< 0.001	1.708 (1.391–2.098)	< 0.001
Gender				
Female	1		1	
Male	1.145 (0.962–1.364)	0.127	1.320 (1.069–1.631)	0.01
Race		0.077		0.144
White	1		1	
Black	1.484 (1.049–2.100)	0.026	1.528 (1.002–2.332)	0.049
Other/unknown	0.954 (0.674–1.349)	0.789	1.017 (0.667–1.552)	0.937
Marital status				
Married	1		1	
Single/other	1.286 (1.095–1.511)	0.002	1.346 (1.103–1.644)	0.004
Tumor grade		< 0.001		< 0.001
G1/2 (well/moderate)	1		1	
G3/4 (poor/undifferentiated)	1.410 (1.176–1.692)	< 0.001	1.520 (1.221–1.893)	< 0.001
Unknown	0.795 (0.643–0.984)	0.035	0.641 (0.482–0.852)	0.002
Histology		0.001		< 0.001
Squamous cell carcinoma	1		1	
Adenocarcinoma	0.730 (0.615–0.867)	< 0.001	0.585 (0.476–0.719)	< 0.001
Unknown	0.896 (0.610–1.315)	0.574	0.602 (0.354–1.023)	0.061
T stage		< 0.001		< 0.001
T1a	1		1	
T1b	1.444 (1.036–2.011)	0.03	1.422 (0.908–2.229)	0.124
T1x	2.056 (1.566–2.697)	< 0.001	2.511 (1.744–3.615)	< 0.001
Time period				
Early (2004–2009)	1		1	
Late (2010–2014)	0.774 (0.645–0.929)	0.006	0.769 (0.615–0.962)	0.021

CI, confidence interval; CSS, cancer‐specific survival; ES, esophagectomy; ET, endoscopic therapy; HR, hazard ratio; OS, overall survival; RT, radiotherapy.

**Table 6:** Multivariable analysis of predictors of OS and CSS

	OS		CSS	
Variable	HR (95% CI)	*P*	HR (95% CI)	*P*
Treatment group		< 0.001		< 0.001
RT	1		1	
ET	0.371 (0.291–0.473)	< 0.001	0.213 (0.147–0.309)	< 0.001
ES	0.305 (0.227–0.408)	< 0.001	0.246 (0.166–0.364)	< 0.001
Age (years)				
Early (75–79)	1		1	
Elderly (> 79)	1.269 (1.072–1.502)	0.006	1.354 (1.095–1.673)	0.005
Tumor grade		0.001		< 0.001
G1/2 (well/moderate)	1		1	
G3/4 (poor/undifferentiated)	1.295 (1.078–1.555)	0.006	1.378 (1.105–1.718)	0.004
Unknown	0.838 (0.671–1.406)	0.118	0.739 (0.552–0.991)	0.043
T stage		0.005		0.041
T1a	1		1	
T1b	1.758 (1.251–2.471)	0.001	1.819 (1.144–2.894)	0.012
T1x	1.322 (0.993–1.759)	0.056	1.396 (0.953–2.043)	0.087
Time period				
Early (2004–2009)	1		1	
Late (2010–2014)	0.820 (0.683–0.986)	0.035	0.852 (0.680–1.066)	0.161
Marital status				
Single/other	1		1	
Married	0.840 (0.713–0.991)	0.038	0.834 (0.671–1.036)	0.101
Histology		0.415		0.954
Squamous cell carcinoma	1		1	
Adenocarcinoma	1.113 (0.925–1.339)	0.255	0.984 (0.783–1.237)	0.89
Unknown	1.220 (0.821–1.811)	0.325	0.920 (0.536–1.581)	0.764
Gender				
Female	*		1	
Male	*		0.992 (0.783–1.258)	0.95

CI, confidence interval; CSS, cancer‐specific survival; ES, esophagectomy; ET, endoscopic therapy; HR, hazard ratio; OS, overall survival; RT, radiotherapy; *No covariates were included.

Undisplayed data revealed that ET and ES groups were significantly higher than those of RT group after PSM (additional file for review but not for publication).

## Discussion

The number of patients with early stage disease (T1a or T1b) has been increasing, especially in Asia, because of dramatic improvements in endoscopic diagnostic modalities. This type of cancer is classified into mucosal carcinoma (T1a), submucosal carcinoma (T1b), and carcinoma in situ (Tis), which is equivalent to stages 0 and l in the TNM classification. We explored the preferred treatment for cT1N0M0 in this study. Although esophagectomy remains the cornerstone of treatment for resectable esophageal carcinoma,^19^ endoscopic methods (i.e. endoscopic mucosal resection [EMR] and endoscopic submucosal dissection [ESD]) have emerged as viable endoscopic options for precise staging and (in select cases) resection of early stage tumors with curative intent. EMR and ESD are appropriate options for patients with node‐negative, small (< 2–3 cm) T1a and low‐risk T1b (e.g. no lymph vascular or SM1 invasion) tumors.^20^, ^21^, ^22^ Elderly patients are generally excluded in most clinical trials, and as a result, the preferred treatment modalities remain unclear.

In this population‐based study, we found that clinical characteristics differed among ES, ET, and RT groups. In the ES group, more patients were younger with better tumor differentiation and T1b staging; in the ET group, more patients were older with T1a staging at a later year of diagnosis; while in the RT group, more patients were older at an early year of diagnosis. The proportion of patients who died of esophageal cancer was significantly higher in the RT than in the ES and ET groups. Our results are consistent with the findings of other research, suggesting that aging, T stage, and tumor differentiation are related to a poor prognosis.^23^, ^24^, ^25^, ^26^

Our results demonstrated that the use of ET increased from 2004–2009 to 2010–2014, while the use of RT has decreased over time, and ES remained relatively stable. It is encouraging that over the study period there was a progressive increase in five‐year OS and CSS. These trends suggest that the growing use of ET did not reduce the long‐term survival of elderly patients with early esophageal cancer. This finding is similar to the results of other studies.^9^, ^13^, ^27^ In recent years, esophageal endoscopic technology has gradually been developed and is now widely used in early cT1N0M0 esophageal cancer patients, but does not affect the CSS of such patients. It is expected that the proportion of patients with early esophageal cancer treated via endoscopic methods will continue to increase.

In our study, all patients were elderly (≥ 75 years). The five‐year OS (ET: HR 0.371, 95% CI 0.291–0.473, *P* < 0.001; ES: HR 0.305, 95% CI 0.227–0.408, *P* < 0.001) and CSS (ET: HR 0.213, 95% CI 0.147–0.309, *P* < 0.001; ES: HR 0.246, 95% CI 0.166–0.364, *P* < 0.001) in the ET and ES groups were significantly higher than in the RT group. Given the younger age of the ES group, we performed PSM and found that the ES group had similar five‐year OS and CSS to the ET group, which was significantly higher than in the RT group. A previous National Cancer Database study analyzed patients (≥ 80 years) with early esophageal cancer and found that patients undergoing ES and ET had similar long‐term survival superior to those treated with chemotherapy or RT.^14^ Another SEER analysis reported similar survival benefits of ES and ET for patients with cT1N0M0 esophageal cancer after PSM.^2^ The results of the present study and previous research strongly support the use of ET as an alternative for the treatment of early esophageal cancer.

We found that long‐term survival in the RT group was significantly lower than in the ES and ET groups. Multivariate analysis also indicated RT as an independent risk predictor for survival. Over the past decade, the use of RT has been decreasing. This trend reflects the poor ability of early esophageal cancer patients to tolerate RT. RT is not suitable for the treatment of elderly patients with early esophageal cancer. A previous study demonstrated that the trend in reduced use of RT would continue because of the related heart and lung toxicity, and poor tolerance in the elderly.^28^ It is expected that the proportion of elderly patients with esophageal cancer administered RT will continue to decline in the future.

Subgroup analyses revealed that five‐year OS and CSS in the ES and ET groups were superior to those in the RT group by T1a and T1b staging, respectively. The T1a patients in the ES group had higher five‐year OS and similar CSS compared to those in the ET group, but T1b patients in the ET group had higher five‐year CSS and similar OS compared to those in the ES group. Meanwhile, the OS rate of the RT group was lower than in the ES and ET groups. Our results may give the reader the impression that five‐year OS is higher in the ES than in the ET group (50.5% vs. 39.6%; *P* = 0.387). This may be because the patients administered ET and RT were older and experienced more common comorbidities. A SEER study and a meta‐analysis both reported that Tis and T1 esophageal cancer patients treated with ES showed higher five‐year OS than those treated with ET.^28^^,^ 29 Our study found that the five‐year OS of patients with T1a treated with ES was higher than those treated with ET. The possible reason is a bias in selection caused by different clinical baseline characteristics in the ET and ES groups. Therefore, we used PSM to adjust baseline covariates and found no difference in five‐year OS and CSS between ES and ET groups with T1a and T1b staging. This conclusion strongly supports the use of ET for the replacement of early esophageal cancer in the elderly. In the present study, 19.2% of the T1b patients treated with ET were older than those in the ES group. The possible reason is that this part of the patient is older than the T1b patients in the ES group and is not in accordance with the standard of surgical treatment. Although the lymph node metastasis rate of submucosal esophageal cancer was 20%, there was no difference in OS and CSS in T1b patients between the ET and ES groups.^30^, ^31^ We consider that ET has satisfactory efficacy and considerable cancer‐induce death compared to ES for elderly patients with esophageal cancer. In the present study, RT outcomes were not consistent with the results of other excellent manuscripts published. We speculate the probable cause as selection bias, as some patients were treated with antiquated radiation techniques and RT alone, not chemoradiotherapy.

There are several advantages to using SEER data for this study. Specifically, large sample sizes and long‐term follow‐up enable reporting of survival outcomes and provide evidence to compare different treatments. The interpretation of our results, however, is restricted by several limitations. Firstly, because of the retrospective nature of the study, patient characteristics were not comparable. T1a and T1b cohorts are different in terms of prognosis and choice of treatment. In addition, the histological types squamous cell carcinoma and adenocarcinoma have different natures in terms of prognosis, proper treatment modality, and response to treatment modality. PSM could not overcome these problems in this study. The current SEER database lacks information on medical history; comorbidities; complications; operation details (open or minimally invasive), medical center information (hospital volume, surgical and endoscopic experience); lymph node involvement; postoperative nutrition status (e.g. hemoglobin); and subsequent therapy (e.g. chemotherapy, hormonal therapy, or biotherapy). Given the similar OS and higher CSS in the ES and ET groups, the lack of such information may not have affected our overall results. Secondly, T1a or T1b was not confirmed pathologically in the ET (57.6%) or ES (28.1%) groups. These patients showed similar long‐term survival and further multivariate analysis concluded the same survival outcomes after these patients were excluded. Additionally, SEER does not provide information on local recurrence or distant metastases. However, we calculated CSS, which may relate to tumor recurrence and metastases. Moreover, as certain comorbidities could have affected the choice of procedure, bias may have affected the analysis. This is another reason for choosing PSM analysis; although PSM analysis may not be a panacea, it can better handle the covariate imbalance. To minimize interference from baseline differences in each treatment group, we used PSM to analyze treatment outcomes and used OS and CSS as the primary treatment outcomes.

Our population‐based study demonstrated better OS and CSS outcomes of ES and ET compared to RT for elderly patients with cT1N0M0 esophageal cancer. ES and ET showed similar survival benefits in all patients and T staging subgroups. Our findings strongly support the use of ET for elderly patients with early esophageal cancer. We suggest that further randomized controlled studies investigate the efficacy of ET rather than ES for the treatment of early esophageal cancer in the elderly.

## Disclosure

No authors report any conflict of interest.
